# The Combined Expression Profiles of Epigenetic Biomarkers Reveal Heterogeneity in Normospermic Human Sperm Samples

**DOI:** 10.3390/genes16111314

**Published:** 2025-11-02

**Authors:** Nino-Guy Cassuto, Florence Boitrelle, Lea Ruoso, Omar Bouattane, Marion Bendayan, Lina Abdiche, Lionel Larue, Gwenola Keromnes, Nathalie Lédée, Laura Prat-Ellenberg, Geraldine Dray, Alexandre Rouen, John De Vos, Said Assou

**Affiliations:** 1 ART Unit, Drouot Laboratory, 75009 Paris, France; 2 Biology-Reproduction-Epigenetic-Environment-Development BREED, INRAE, Paris Saclay University, UVSQ, 78350 Jouy-en-Josas, France; 3 ENSET Mohammedia, Hassan II University of Casablanca, Casablanca 21100, Morocco; 4 IVF ART Diaconesses-Drouot Hospital, 75012 Paris, France; 5 IVF Center Bluets-Drouot, Les Bluets Hospital, 75012 Paris, France; 6 Unité de Formation et Recherche (UFR), University of Versailles-St Quentin en Yvelines (UVSQ), 78035 Versailles, France; 7 VIFASOM, Université Paris Cité, 75006 Paris, France; 8 Institute for Regenerative Medicine and Biotherapy (IRMB), University of Montpellier, INSERM, CHU Montpellier, 34295 Montpellier, France

**Keywords:** gene expression, biomarkers, sperm function, early embryogenesis, male infertility

## Abstract

**Background:** Male infertility is evaluated using standard semen parameters. However, these criteria offer limited insight into sperm functionality and poorly predict natural fertility or assisted reproductive technology (ART) outcomes. **Methods:** In this study, the expression levels of three genes (*AURKA*, *HDAC4*, and *CARHSP1*) involved in mitosis regulation, epigenetic modulation and early embryonic development, were measured by RT-qPCR in sperm samples (training dataset). For each gene, thresholds of normal and reduced expression were established by biostatistical modeling and combined with the number of motile spermatozoa to develop the Spermatozoa Function Index (SFI). **Results:** The ROC analysis was used to interpret the SFI values: SFI > 320 (normal), 290–320 (intermediate), and <290 (low). Then, this index was validated using 627 fresh semen samples from 25- to 60-year-old men at our ART center. Based on the World Health Organization criteria, 54.5% of the 627 sperm samples were normospermic, 8.8% showed oligo-astheno-teratospermia, and 36.6% had one or two abnormal parameters. According to the SFI values, 41% of sperm samples displayed normal expression, 55.9% low expression, and 4.1% intermediate expression. Only 57% of the 342 normospermic samples had normal SFI values and 37% had low SFI values. Among the 81 samples with stringent normal criteria (≥50 million/mL, ≥50% total motility, ≥14% normal morphology), 67.9% displayed normal SFI and 22.2% low SFI values. These findings suggest that even sperm with normal parameters may harbor dysfunctions. **Conclusions:** Our data highlight a gene signature with strong discriminatory power and promising diagnostic value for detecting subclinical sperm defects and improving male infertility assessment.

## 1. Introduction

The long-standing dogma that spermatozoa are transcriptionally silent cells solely dedicated to delivering the paternal DNA during fertilization is no longer valid. Human spermatozoa are now considered highly specialized cells with remarkable, sophisticated molecular complexity that play a crucial role in fertilization and early embryonic development. Despite their tightly compacted chromatin and minimal transcriptional activity, they retain a rich and functionally relevant RNA repertoire that includes coding mRNAs and various non-coding RNA types, such as microRNAs, tRNA-derived fragments, piRNAs, and long non-coding RNAs [1]. Sperm RNA amount and composition can influence early embryonic development and may be altered in men with infertility [2]. This transcriptome, largely inherited from the earlier stages of spermatogenesis, provides key insights into male reproductive biology and male fertility regulation [3]. Moreover, sperm RNAs and epigenetic marks are increasingly implicated in early embryonic development, highlighting a more active and nuanced role for spermatozoa in post-fertilization processes [4,5,6]. Sperm goes far beyond its role in fertilization and clinical studies confirm that sperm function is a crucial aspect of research to increase the efficacy of assisted reproduction treatments (ART).

Recent advancements in molecular biology and translational research have significantly expanded our understanding of the complex molecular mechanisms underlying male fertility. The advent of high-throughput technologies and multi-OMICS platforms has provided unprecedented insights into the molecular orchestration of spermatogenesis and sperm function [7]. In this highly coordinated system, the different OMICS layers operate in concert to maintain the male reproductive capacity. A recent large-scale longitudinal study spanning five decades and involving 78,284 men identified semen quality as a strong biomarker of general health and longevity, revealing that men with better semen parameters tend to have a significantly longer life expectancy [8]. In parallel, many studies reported a global decline in sperm counts and a reduced proportion of morphologically normal spermatozoa in the past decades [9,10], raising concerns about reproductive and general male health [11].

The first step in the evaluation of male fertility is the semen analysis, according to the World Health Organization (WHO) criteria [12]. This analysis assesses key parameters, such as sperm concentration, motility and morphology [13]. This method allows classifying sperm samples into different categories (normospermia, oligospermia, asthenospermia, and aspermia), but it remains insufficient for accurately predicting natural conception, fertilization potential, and ART outcomes. New tests are needed to predict the success and outcomes of in vitro fertilization [14]. Consequently, there is a growing demand for integrative approaches that combine morphological evaluation and molecular and functional profiling, to improve diagnostic precision and guide personalized therapeutic strategies.

In a previous study, we described a high-resolution, dynamic scoring system of sperm head morphology based on strict criteria that correlates with blastocyst expansion on day 5 post-fertilization [15]. This scoring system assesses sperm quality based on head shape and size, basal morphology, and the presence/absence of vacuoles. Scores range from 6 (high-quality) to 0 (low-quality). A higher score signifies normal nuclear morphology, absence of vacuoles, and well-defined basal structures. Independent studies confirmed the positive correlation between higher scores and improved fertilization and blastulation rates [16,17,18]. Moreover, we performed a whole-genome sequencing analysis to compare high-quality (score 6) and low-quality (score 0) spermatozoa [19]. This analysis revealed marked differences in DNA methylation, highlighting a distinct epigenetic regulation. Such differential methylation patterns indicate that spermatozoa in a single sample can differ in reproductive competence (i.e., fertilization, embryo development, and pregnancy outcomes). In addition, testis-specific microRNAs appear to play a central role in orchestrating this regulation [20]. These findings are consistent with previous evidence that normal spermatozoa with and without vacuoles have different epigenetic profiles and that the selection of those without vacuoles promotes embryo development and offspring outcomes [21]. Building on these insights, the present study focused on three candidate genes (*AURKA*, *HDAC4*, and *CARHSP1*) as molecular biomarkers of sperm quality. Indeed, these genes converge on key biological pathways identified in our earlier epigenetic and network analyses. *AURKA*, a master regulator of the cell cycle, interacts with *HDAC4*, a chromatin acetylation modulator. *CARHSP1* links calcium signaling to sperm function. All three genes are strongly expressed in morphologically normal spermatozoa, supporting their role as positive markers of functional competence. Their testis-enriched expression profiles, Gene Ontology (GO) annotations, and functional relevance further emphasize their potential value as biomarkers of spermatogenesis and sperm function [19].

Here, the expression of *AURKA*, *HDAC4*, and *CARHSP1* was measured by RT-qPCR in sperm samples stratified according to our high-resolution dynamic scoring system (score 6 versus score 0). For each gene, biostatistical modeling was applied to establish thresholds of normal vs. reduced expression. Then, their expression values were integrated into a composite Spermatozoa Function Index (SFI) that included also the number of motile spermatozoa and that was validated in a cohort of men enrolled at our ART center. The combination of the expression data of the three genes into a unique molecular signature (SFI) gave a test with high discriminatory power, enabling a more robust and clinically relevant evaluation of sperm functionality. Overall, this strategy facilitates the identification of transcriptomic and epigenetic profiles in sperm that are predictive of its fertility potential.

## 2. Materials and Methods

### 2.1. Sample Collection and Preparation

The study was approved by the Institutional Review Board (IRB) of the French Language Andrology Society (IORG0010678) and conducted at the ART Unit of the Drouot Laboratory, Paris, France. All participants provided a written informed consent prior to inclusion and were informed that their semen samples (what left after routine clinical testing) would be analyzed using high-magnification microscopy and molecular biology techniques. This procedure did not affect their treatment. No financial compensation was provided, and all data were anonymized.

### 2.2. Semen Collection and Analysis

The study included 627 fresh ejaculates from 20 to 60-year-old men who attended our ART center for routine semen analysis between March 2024 and July 2025. Inclusion criteria were negative serology for human immunodeficiency virus (HIV), hepatitis B virus (HBV), hepatitis C virus (HCV) and syphilis. Samples of testicular or epididymal origin, and samples with cryptospermia or severe oligospermia (<0.5 million/mL) were excluded. Participants were predominantly categorized as Caucasian (72%), followed by African (24%) and Asian (4%); 2.61% had a medical history affecting the male reproductive system and 20.15% were smokers.

Semen samples were obtained by masturbation on-site and analyzed within 30 to 60 min after ejaculation. Standard semen parameters (volume, concentration, motility and morphology) were evaluated manually (spermiogram) and also using a semi-automated spermocytogram (Dimension II, Hamilton Thorne, version 3.2.8, COFRAC-accredited) following the 6th edition of the *WHO Laboratory Manual for the Examination and Processing of Human Semen* (World Health Organization, Geneva, 2021) [12]. The study included semen samples that covered the full spectrum of WHO parameters, from completely normal (sperm concentration, progressive motility, and morphology) to samples with one, two, or three abnormal parameters. Motile spermatozoa were isolated and purified using a bilayer density gradient consisting of 90% and 45% Isolate Sperm Separation Medium (Cat. no. 99264; Fujifilm Irvine Scientific, Santa Ana, CA, USA) in conical tubes. Following centrifugation at 300× *g* for 15 min, the supernatant was discarded, and the sperm pellets were washed in modified Human Tubal Fluid (mHTF) medium (Cat. no. 90126; Fujifilm Irvine Scientific, Santa Ana, CA, USA). A second centrifugation was performed at 600× *g* for 10 min, after which pellets were resuspended in 300 μL of mHTF medium. This double-gradient centrifugation protocol allows removing somatic cells, leukocytes and immotile spermatozoa. Then, after sperm concentration and motility analysis, samples were stored at −80 °C. Rapid freezing of the purified sperm pellets immediately after preparation helps to preserve RNA integrity.

### 2.3. RNA Extraction and RT-qPCR

Total RNA was extracted from frozen sperm pellets (300 μL) using the Maelstrom 9600 system (TANBead) and the OptiPure Viral Auto Plate kit (REF: W665A46), following the manufacturer’s instructions. Complementary DNA (cDNA) synthesis and quantitative PCR (qPCR) were performed on a CFX96™ Real-Time PCR Detection System (Bio-Rad, Hercules, CA, USA). The thermal cycling protocol consisted of an initial reverse transcription at 45 °C for 5 min, followed by denaturation at 98 °C for 20 s. Amplification was carried out in 45 cycles, each comprising denaturation at 98 °C for 3 s and annealing/extension at 60 °C for 10 s, and fluorescence acquisition in the extension phase. The quadruplex real-time PCR assay targeted the three genes of interest (*AURKA*, *HDAC4* and *CARHSP1*) and a housekeeping gene. Gene-specific detection was achieved using probes labeled with the FAM, HEX, Texas Red, and Cy5 fluorophores. For each PCR run, efficiency was monitored on standard curves using 10-fold serial dilutions (from 10^5^ to 10^2^ copies per µL) of four control plasmids that contained the target sequences. PCR efficiency was consistently between 90% and 110% in all runs. The expression levels (copies per µL) were obtained for each target using the standard curves and were then normalized by dividing the number of copies per µL of the target RNA by the number of copies per µL of the housekeeping gene [22].

### 2.4. Data Preparation and Modeling.

The target variable (high-resolution dynamic score) was binarized (0 for score 0, 1 for score 6), resulting in a dataset the included three predictor variables (*AURKA, HDAC4* and *CARHSP1*) and the target variable. The dataset (*n* = 106) was randomly split into training (70%) and test (30%) sets, and predictors were standardized using z-scores. A logistic regression model was trained on the standardized data, and performance was evaluated in both training and test sets, showing high accuracy and recall, with no evidence of overfitting. The relative contribution of each predictor to the model was quantified with odds ratios. This revealed that one variable (*HDAC4*) had a predominant effect in predicting a score 6. To optimize the classification, the decision threshold was determined using a precision–recall curve, balancing false positive and true positive detection. An optimal threshold of 0.35–0.38 was identified that improved the model performance: in the training set, precision and recall were ~96% and 92%, respectively, and in the test set they reached 100%, confirming the model robustness and predictive accuracy.

### 2.5. Protein Localization and RNA Expression

The protein expression and localization of AURKA, HDAC4, and CARHSP1 in human testis samples were examined using immunohistochemistry (IHC) data from the Human Protein Atlas (HPA, *https://www.proteinatlas.org/*). Images of formalin-fixed, paraffin-embedded normal testis tissues from adult donors were generated by the HPA consortium using antibodies validated for IHC (against AURKA: CAB001454; against HDAC4: HPA071448; against CARHSP1: HPA051911). Our analysis used data from six adult male donors (from 25 to 56 years of age) and all tissue samples were collected in accordance with the ethical guidelines for human biobanks and genetic research. In stained tissue sections, expression was analyzed in germ cells and somatic cells, and localization was annotated according to the HPA database, which integrates IHC, bulk transcriptomics and single-cell data [23]. To assess tissue-specific expression, transcriptomic data from the Genotype Tissue Expression (GTEx) project were retrieved via the HPA portal. The normalized transcript levels (nTPM, normalized transcripts per million) of *AURKA*, *HDAC4* and *CARHSP1* were extracted for different human tissues and compared between testis and other organs. At the single-cell level, RNA expression patterns were examined using single-cell RNA sequencing data of adult human testis samples from the *UCSC Cell Browser* (https://cells.ucsc.edu/?ds=adult-testis, accessed on 17 May 2025), based on the dataset [24]. Testicular cell populations were profiled at the single-cell resolution and visualized with UMAP plots, where each dot represents an individual cell and color intensity reflects gene expression. Expression of *AURKA*, *HDAC4* and *CARHSP1* was mapped in germ cell and somatic cell populations to evaluate cell-type specificity during spermatogenesis. The GeneMANIA database was used to predict the function of *AURKA*, *HDAC4* and *CARHSP1* (https://genemania.org/).

### 2.6. Statistical Analysis

Statistical analyses were performed using GraphPad Prism (version 10.5.0) and Python libraries (version 3.10). Data distribution was first assessed using the Shapiro–Wilk test. Differences between two groups were analyzed with the unpaired two-tailed Student’s *t*-test, and results are expressed as mean ± standard deviation (SD). Correlations between variables were evaluated using Pearson’s correlation coefficient. A *p*-value < 0.05 was considered statistically significant.

## 3. Results

### 3.1. Relevance of Candidate Gene Expression Patterns for Testis-Specific Biomarker Identification

**Figure 1:**
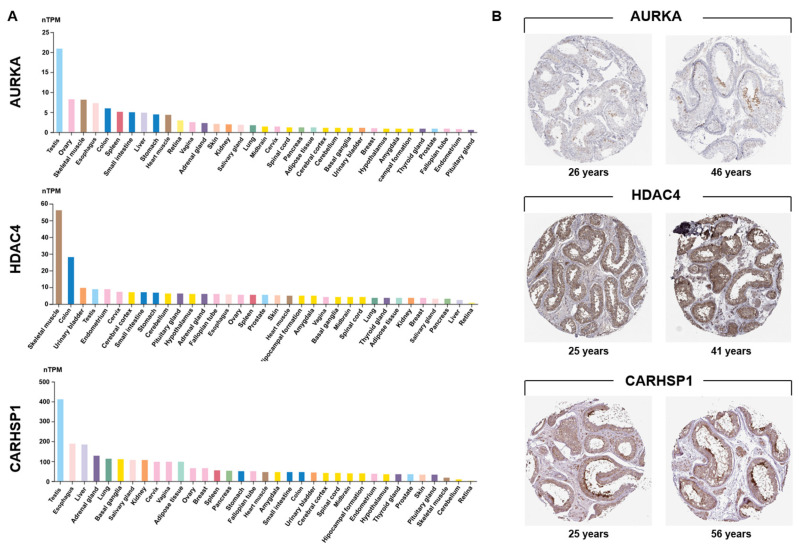
Testis-specific expression of *AURKA*, *HDAC4* and *CARHSP1*. (**A**) Analysis of GTEx transcriptomic data revealed a marked testis-enriched expression pattern for the three candidate genes. Expression values (in TPM) of *AURKA*, *HDAC4* and *CARHSP1* in 35 human tissues from the GTEx consortium (Human Protein Atlas). For each gene, the colored histogram represents the mean TPM value of all samples for that tissue. nTPM: normalized transcripts per million. (**B**) Immunohistochemical localization of AURKA, HDAC4, and CARHSP1 in human normal testis sections from adult donors of different ages. AURKA shows predominant expression in pachytene spermatocytes. HDAC4 is strongly detected in cells of the seminiferous tubules and Leydig cells. CARHSP1 expression is observed in elongated/late spermatids, pachytene spermatocytes and Leydig cells. Images were obtained from the Human Protein Atlas (Testis (T-78000), Patients id (2071, 2435, 3627, 4485, 3627, 3355)).

*AURKA*, *HDAC4* and *CARHSP1* were identified in our previous whole genome sequencing study as differentially methylated and differentially expressed genes in high-quality (score 6) vs. low-quality (score 0) spermatozoa [19], suggesting a potential involvement in male fertility (GO analysis). Integrative transcriptomic and proteomic analyses consistently predicted that these three genes are strong candidate biomarkers of testis function. Transcriptome profiling using data from the GTEx database confirmed that their expression was enriched in testis compared with most other organs (Figure 1A). *AURKA* was strongly expressed in testis, and at moderate levels in hematopoietic and lymphoid tissues, consistent with a specialized role in meiotic progression. *CARHSP1* displayed a striking testis-specific enrichment, underscoring its potential as a germ cell–specific regulator. *HDAC4* also was expressed in testis, albeit at lower levels than in skeletal muscle. However, its expression in different tissues of the reproductive system suggested regulatory functions in germ cells and somatic cells. Immunohistochemistry data from the Human Protein Atlas confirmed their distinct localization within the seminiferous tubules (Figure 1B).

**Figure 2:**
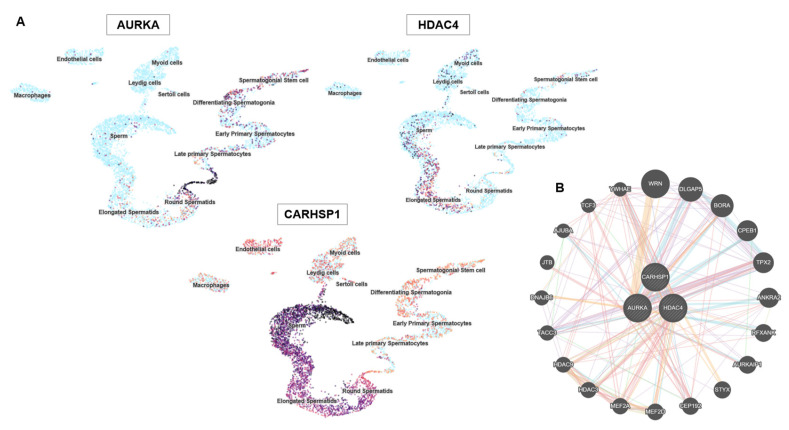
*AURKA*, *HDAC4* and *CARHSP1* gene expression in specific testis cell types and protein interactions. (**A**) UMAP plots showing the single-cell expression patterns of *AURKA*, *HDAC4* and *CARHSP1* in the testis. Each point represents a single cell and clustering reflects similarity in gene expression. The color intensity indicates the normalized expression levels (dark = high, light = low). *AURKA* is strongly expressed in proliferating cells, including spermatogonial stem cells and differentiating spermatogonia. *HDAC4* shows diffuse expression in multiple cell types, including Sertoli and Leydig cells. *CARHSP1* expression is restricted to late primary spermatocytes and differentiating spermatogonia, highlighting stage-specific roles during spermatogenesis. (**B**) Protein–protein interaction network of AURKA, HDAC4 and CARHSP1 generated using the GeneMANIA database.

AURKA was confined to pachytene spermatocytes, highlighting its meiotic role. HDAC4 was detected in sperm cells within the seminiferous epithelium and in Leydig cells, suggesting dual germline and somatic regulatory activity. CARHSP1 was broadly expressed at several spermatogenesis stages (including pachytene spermatocytes, late spermatids) and also in Leydig cells, indicating a wider contribution to spermatogenesis. Single-cell RNA sequencing data [24] analysis refined these patterns: *AURKA* was enriched in late primary spermatocytes and round spermatids; *HDAC4* was expressed in early germ cell populations and somatic (Sertoli and Leydig) cells; and *CARHSP1* was predominantly expressed in round and elongated spermatids, and also in spermatocytes (Figure 2A). Altogether, these data demonstrate that *AURKA*, *CARHSP1* and *HDAC4* are consistently and specifically expressed in key germ cell subtypes of the testis. Moreover, these genes were interconnected within a functional network (Figure 2B). Their stage-dependent and cell-type–restricted expression strongly supports their selection as promising biomarkers of spermatogenesis and male reproductive potential.

### 3.2. Establishment of the Spermatozoa Function Index (SFI)

**Figure 3:**
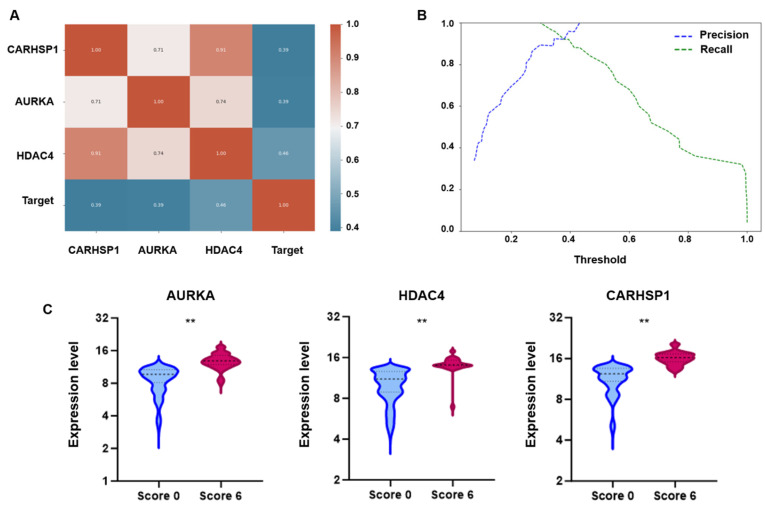
Correlation, classification performance, and differential expression analysis of candidate genes. (**A**) Pearson correlation heatmap. The heatmap displays the Pearson correlation coefficients between the three genes (*AURKA*, *HDAC4*, and *CARHSP1*) and the binary target variable (Target; high-resolution dynamic score). Coefficients range from −1 to 1; values close to 1 indicate strong positive correlation, values close to −1 indicate strong negative correlation, and values near 0 reflect no linear correlation. (**B**) Precision–recall analysis of the logistic regression model illustrates the effect of varying the decision threshold on the classification performance using the SFI. At very low thresholds, recall was close to 1, indicating that nearly all true positives were detected, but precision remained low due to a high number of false positives. Conversely, as the threshold approached 1, precision increased while recall decreased, reflecting a trade-off between the two metrics. An optimal balance was identified at 0.35–0.38, where both precision and recall were high, providing the best compromise for accurate classification. This threshold yielded improved performance in both training and test sets compared to the conventional 0.5 cut-off, confirming its relevance for robust sample discrimination. (**C**) Box plots showing the expression levels of *AURKA*, *HDAC4* and *CARHSP1* in sperm samples stratified in function of their high-resolution dynamic score (Score 0 vs. Score 6). For all three genes, expression was significantly higher in the Score 6 group than the Score 0 group (** = *p* < 0.01).

To develop the SFI, the expression levels of *AURKA*, *HDAC4*, and *CARHSP1* were assessed in a dataset of 106 sperm samples (based on the high-resolution dynamic scoring system). First, the correlations among the three genes and their relationship with the target variable (i.e., the high-resolution, dynamic score: 6 vs. 0) were evaluated. The correlation matrix, displayed as a heatmap (Figure 3A), highlighted strong positive correlations among all three genes. *HDAC4* expression showed the strongest association with the binary target variable. This suggests that although the three genes are interrelated, *HDAC4* plays a more prominent role in distinguishing samples according to their score. Moreover, a logistic regression model trained using the z scores of the expression data could classify sperm samples effectively based on the three gene expression profiles. The analyses using the training and test datasets confirmed its predictive power. The correlation and predictive weight of *HDAC4* were particularly high, reinforcing its potential as a key biomarker. To refine the classification, precision–recall curves were generated, and the decision cut-off was optimized to balance sensitivity and specificity. Selecting an optimal threshold of 0.35–0.38 (Figure 3B), instead of relying on the conventional 0.5 cut-off, substantially improved the predictive performance, which is crucial in a biomedical application context. Based on these results, the individual gene expression profiles were combined into a composite index (SFI), defined as: SFI = (3.14 · Δ[RNA] (CARHSP1) + 4.51 · Δ[RNA] (AURKA) + 18.91 · Δ[RNA] (HDAC4)) · (s · v ·1000/300), where Δ[RNA] represents the ratio of target RNA molecules to housekeeping RNA molecules, *s* is the number of motile sperm cells in 0.3 mL of sample, and *v* is the total volume of the extracted sample. Lastly, SFI thresholds were derived from the training dataset to classify sperm functionality: SFI values > 320 defined normal functionality (normal expression), <290 low functionality (low expression) and 290–320 an intermediate functionality (intermediate expression) (Figure 3C). These thresholds were then tested in an prospective cohort of 627 semen samples, to validate SFI robustness as a sperm functional biomarker.

### 3.3. Comparison of WHO Semen Criteria and SFI in 627 Sperm Samples

**Figure 4:**
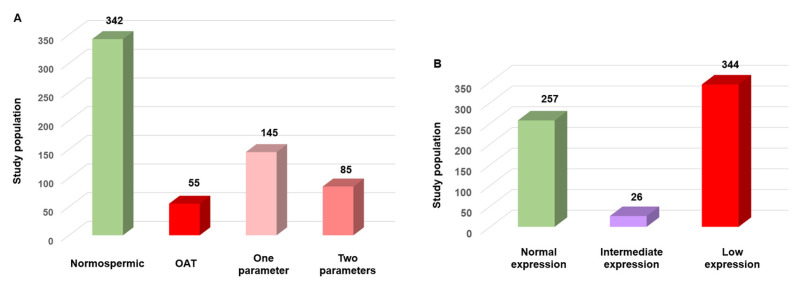
Comparison of semen quality using the WHO criteria and the SFI in 627 human sperm samples. (**A**) Sample distribution according to the WHO criteria: 54.5% normospermic, 23.1% with a single altered parameter, 13.5% with two altered parameters, and 8.8% with oligo-astheno-teratospermia (OAT). (**B**). Sample distribution based on the SFI: 54.9% with low expression, 41% with normal expression (SFI > 320), and 4.1% with intermediate expression.

To validate SFI robustness as a sperm functional biomarker, 627 human semen samples were analyzed using the standard WHO criteria and the new SFI. Based on the WHO reference criteria, 342/627 samples (54.5%) were classified as normospermic, 55 samples (8.8%) showed oligo-astheno-teratospermia with concurrent abnormalities in concentration, motility and morphology, and 230 samples (36.6%) presented one (*n* = 145; 23.1%) or two (*n* = 85; 13.5%) altered parameters (Figure 4A). Based on their SFI value, 344/627 (54.9%) samples had low expression (SFI < 290), 26 samples (4.1%) were in the intermediate category and 257 samples (41%) had normal expression (SFI > 320) (Figure 4B).

### 3.4. SFI in Normal Sperm Samples According to the WHO Criteria

**Figure 5:**
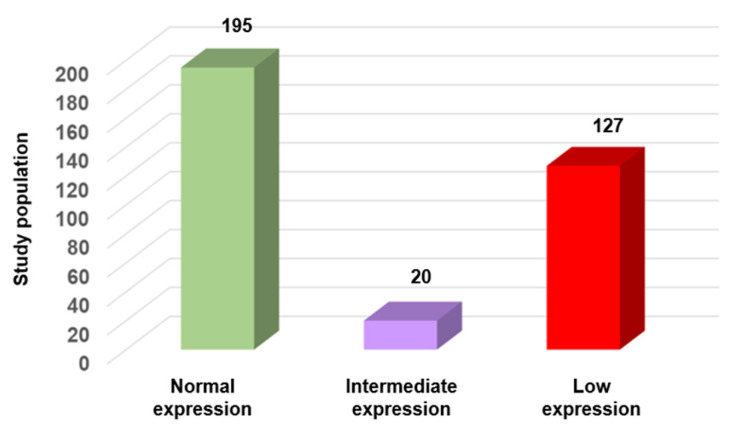
SFI variation in normal sperm samples according to the WHO criteria (*n* = 342). Samples were categorized based on their SFI value into three groups: normal expression (*n* = 195; 57%), intermediate expression (*n* = 20; 5.8%), and low expression (*n* = 127; 37%).

According to the WHO criteria (concentration, progressive motility, and morphology) 342/627 sperm samples (54.5%) were normospermic. However, their SFI values revealed substantial heterogeneity within this “normal” group. Specifically, SFI was >320 (normal expression/functionality) and between 290 and 320 (intermediate expression/functionality) only in 195/342 (57%) and 20/342 (5.8%) samples, respectively. Conversely, SFI was <290 (low expression/functionality) in 127 (37%) samples that were considered normal according to the WHO criteria (Figure 5), but in which the expression of three key genes involved in sperm function was reduced. These findings emphasize the molecular heterogeneity within sperm populations that are considered normal. Such differences, undetectable by standard parameters, may contribute to unexplained infertility or poor ART outcomes.

### 3.5. SFI Variations in High-Quality Sperm Samples

**Figure 6:**
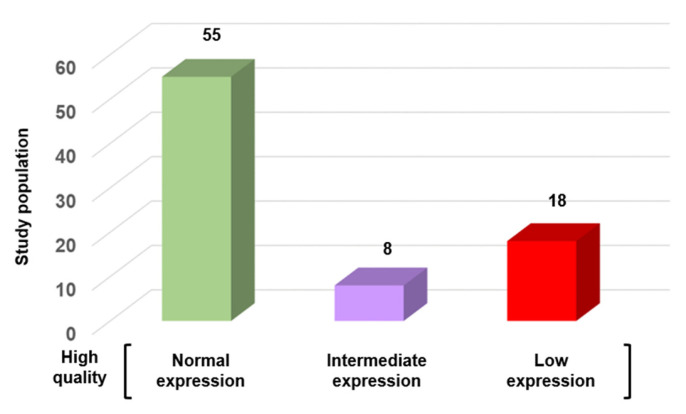
SFI variation in high-quality sperm samples according to the WHO criteria (*n* = 81). Samples were categorized based on their SFI value into three groups: normal expression (*n* = 55; 67.9%), intermediate expression (*n* = 8; 9.9%), and low expression (*n* = 18; 22.2%).

To examine the relationship between conventional sperm quality criteria and SFI, a subgroup of 81 “very high-quality” sperm samples was used (22.7% of the 342 samples with normospermia). These samples met the WHO criteria and were in the upper quartile for concentration (≥50 million/mL), total motility ≥ 50%), progressive motility (≥40%), and normal morphology (≥14%, Kruger’s strict criteria). Even in this subgroup, SFI was >320 only in 55 (67.9%) samples. The other samples had an intermediated SFI (*n* = 8; 9.9%) or even low SFI (*n* = 18; 22.2%) value (Figure 6). This discordance highlights the limitations of routine semen analysis in detecting molecular or epigenetic abnormalities that may influence fertilization, embryo development, or pregnancy outcomes.

Deficiencies in these molecular layers, undetectable through standard morphological and kinetic assessments, may contribute to unexplained infertility or repeated ART failures. Collectively, these results underscore the need to complement conventional semen analysis with molecular diagnostic tools.

## 4. Discussion

In the past decade, attempts to optimize sperm selection for ART have mainly focused on conventional semen parameters, such as concentration, motility, and morphology. Indeed, male infertility is routinely assessed through a visual examination of these parameters. These criteria provide information on sperm quality, but their normality does not indicate the complete absence of molecular alterations. Particularly, the transcriptomic and epigenetic landscapes of spermatozoa, which are critical for fertilization competence and early embryonic development, remain largely neglected in routine andrological assessments. This could be explained by the fact that for long time spermatozoa were considered just the passive transporters of the paternal genome. However, accumulating evidence highlights their active regulatory role in embryonic programming and reproductive success [5,25,26,27]. Moreover, several sperm proteins have emerged as potential biomarkers that could predict the outcome in ART, leading to the possibility of improved diagnosis.

These findings emphasize the urgent need of more comprehensive molecular sperm studies to develop diagnostic tools for assessing the roles of the transcriptomic profile and DNA methylation variability in sperm cells in the fertility potential. Our study identified three testis-enriched genes (*AURKA*, *HDAC4* and *CARHSP1*) as potential biomarkers of spermatogenesis and sperm function. *AURKA* plays a pivotal role in spindle assembly during meiosis and early embryonic mitotic division. Arrested embryos display transcriptome alterations that are coordinated with multiple epigenetic reprogramming defects, and also *AURKA* downregulation [28]. *HDAC4* is a key epigenetic regulator that influences chromatin remodeling and histone-to-protamine exchange, essential for DNA compaction and genomic stability. Its deregulation contributes to epigenetic transmission defects and to a pathological mechanism with uncharacterized developmental disorder [29]. *CARHSP1* (also called *CRHSP-24*), a stress-responsive RNA-binding protein, maintains transcriptomic integrity during spermiogenesis and stabilizes mRNAs through transcriptionally silent phases of sperm maturation [30,31]. Our study shows that these three genes are strongly expressed in germ cells (GTEx and human testis datasets) and our previous work highlighted that they were among the genes with differential DNA methylation in sperm samples with score 6 vs. score 0 [19]. Additionally, post-transcriptional regulation by microRNAs, including miR-34c and miR-125a, modulates their expression during late spermatogenesis and fertilization. Our recent study further supports the functional relevance of these microRNAs in human sperm [20].

Then, the expression levels of *AURKA*, *HDAC4* and *CARHSP1* were used to develop the SFI that revealed substantial heterogeneity even among sperm samples considered morphologically normal and of “very high quality” according to the WHO criteria. A considerable proportion of normospermic samples exhibited low SFI value as well as 22% of “very high quality” samples, reflecting hidden transcriptomic alterations. This suggests that conventional semen parameters do not equate to intact transcriptomic profile. These findings corroborate previous studies demonstrating poor correlation between standard semen metrics and fertilization outcomes, particularly in unexplained infertility, and are consistent with emerging evidence from proteomic and epigenetic analyses. The present study focused on extreme categories (6 vs. 0) to capture two clinically distinct phenotypes and maximize the discriminative power of the model. This approach may not directly capture intermediate phenotypes, but it allowed us to robustly identify patterns associated with the most distinct functional outcomes. Class distribution was addressed through stratified cross-validation and loss weighting to ensure reliable performance.

**Figure 7:**
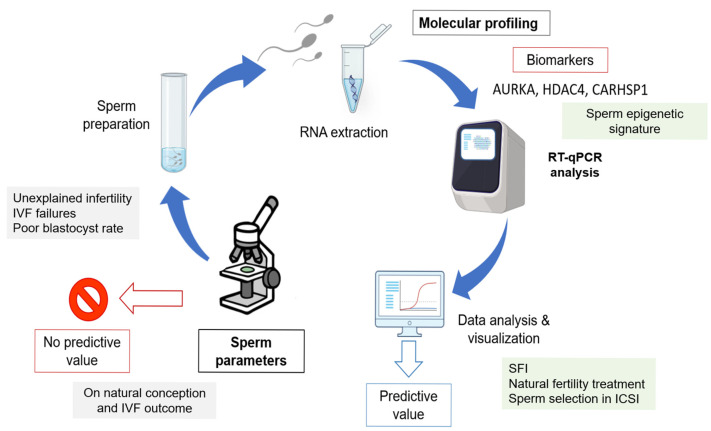
Overview of the molecular strategy to identify spermatozoa with the most favorable gene expression profiles in relation to sperm quality parameters. This approach illustrates how integrating molecular biomarkers with conventional semen parameters could help to discriminate between functionally competent and impaired spermatozoa, thereby improving the male fertility potential assessment.

Clinically, the SFI provides a refined framework to stratify male fertility beyond conventional assessments. By integrating a three-gene expression signature with classical semen parameters, it could contribute to the detection of subclinical dysfunctions that may compromise fertilization or early embryogenesis. The optimization of the SFI classification thresholds enhanced its sensitivity and specificity, addressing the risk of false negatives in clinical practice. Importantly, this approach aligns with the Paternal Origins of Health and Disease (POHaD) paradigm, highlighting that sperm molecular quality affects the immediate reproductive outcomes, but may also influence the offspring long-term health through epigenetic inheritance [21]. Altogether, our results indicate that need of other tests in addition to the WHO parameters to evaluate sperm functionality and support the integration of molecular diagnostic tools, such as transcriptomic profiling and the SFI, into the routine fertility assessment. Such strategies have the potential to enhance diagnostic precision, uncover latent sperm defects, guide personalized interventions, and contribute to improving ART outcomes while providing insights into the broader implications of sperm molecular integrity for the reproductive and offspring health (Figure 7).

Several tests have been developed to complement the standard semen analysis, including the sperm chromatin structure assay, DNA fragmentation index [32] TUNEL assay [33], fluorescence in situ hybridization [34] karyotype analysis [35], and Y-chromosome azoospermia factor microdeletion testing [36]. These tests primarily evaluate sperm DNA integrity or chromosome composition, providing important insights into sperm quality. However, they do not necessarily capture the functional heterogeneity among spermatozoa, particularly in men with normospermia. The SFI, based on the combined expression of *AURKA*, *HDAC4* and *CARHSP1*, offers a complementary perspective by integrating multiple molecular pathways relevant to sperm function. The SFI can identify subtle variations in sperm functional competence that may not be detected by DNA-based or cytogenetic assays, highlighting its potential value as an adjunct tool in fertility evaluation. A detailed comparison of these assays and their complementarity with the SFI is provided in Supplementary Table S1.

Spermatogenesis is a highly complex process that requires the coordinated activity of different cell types, hormones, paracrine factors, genes, and epigenetic regulators [37]. Our findings indicate that *AURKA*, *HDAC4* and *CARHSP1* are interconnected within the same functional network and play roles in key steps of spermatogenesis. Therefore, they may influence sperm morphology, concentration, and motility. This network-based perspective suggests that abnormalities (e.g., altered morphology, oligospermia or asthenospermia) may arise from disruptions in shared molecular pathways, although their severity or clinical presentation may differ. The SFI could capture these interconnected functional impairments, but more studies are needed to determine whether specific molecular signatures correspond to distinct semen phenotypes.

There is growing evidence that lifestyle-related factors such as obesity, tobacco use, and the presence of varicocele may influence sperm epigenetics and thereby contribute to male infertility. Epigenetic alterations, including changes in DNA methylation, histone modifications, and small non-coding RNAs, have been linked to paternal obesity and adverse reproductive outcomes, with potential transgenerational effects [38,39]. Similarly, cigarette smoking has been associated with impaired semen parameters and is suspected to exert epigenetic modifications through oxidative stress and DNA damage pathways. Varicocele, as well as broader lifestyle factors such as diet and alcohol consumption, may also contribute to disrupted sperm function, although their specific epigenetic consequences remain less well characterized [40]. In our study, patients were enrolled randomly without specific assessment of these risk factors, and only conventional sperm parameters were considered. Future studies integrating epigenetic profiling are warranted, as they may reveal novel biomarkers of male infertility and improve our understanding of how modifiable exposures shape reproductive health.

## 5. Conclusions

Our findings demonstrate that traditional semen analysis lacks the fine resolution to detect critical transcriptomic alterations that compromise sperm function. The SFI provides a biologically meaningful and clinically actionable tool to detect these hidden alterations in spermatozoa, offering a refined assessment of the male fertility potential. Its integration into routine fertility evaluations could allow: (i) identifying patients who may benefit from a specific ART treatment and advanced sperm selection protocols; (ii) guiding personalized interventions that target lifestyle and environmental risk factors; and (iii) improving prognostic accuracy in idiopathic and unexplained infertility, ART failures, and poor blastocyst rate. Future work should focus on validating these transcriptomic markers in multicenter studies to enhance sperm selection. Ultimately, systematic molecular profiling of spermatozoa has the potential to improve ART outcomes, inform preventive strategies, and support a deeper understanding of paternal contributions to early development and offspring health and will help to facilitate the management of men with infertility.

## 6. Patents

G.N.C filed a patent entitled “method for selecting spermatozoa, in particular for medically assisted procreation” (priority number: EP2021/052041).

## Data Availability

The original contributions presented in this study are included in the article/Supplementary Materials. Further inquiries can be directed to the corresponding authors.

## Supplementary Materials

The following supporting information can be downloaded at: https://www.mdpi.com/article/10.3390/genes16111314/s1, Table S1: Comparative overview of sperm diagnostic tests and complementarity with SFI.

## Abbreviations

The following abbreviations are used in this manuscript:

ART	Assisted reproductive technology
AZF	Azoospermia factor
COFRAC	Comité français d’accréditation
DFI	DNA fragmentation index
DMCs	Differentially methylated CpGs
FISH	Fluorescence in situ Hybridization
GO	Gene ontology
GTEx	Genotype tissue expression
ICSI	Intracytoplasmic sperm injection
IRB	Institutional review board
IHC	Immunohistochemistry
IUI	Intra uterine insemination
IVF	In vitro fertilization
HBV	Hepatitis B virus
HCV	Hepatitis C virus
HIV	Human immunodeficiency virus
mHTF	modified human tubal fluid
nTPM	Normalized transcripts per million
TUNEL	Terminal deoxynucleotidyl transferase dUTP Nick-End Labeling
OAT	Oligo astheno teratozoospermia
POHaD	Paternal origins of health and disease
SCSA	Sperm chromatin structure assay
SFI	Sperm functional index
WHO	World health organization
